# Effects of remote ischemic preconditioning in severe traumatic brain injury: A single-center randomized controlled trial

**DOI:** 10.1097/MD.0000000000035190

**Published:** 2023-09-22

**Authors:** Juan Shen, Lin Zhu, Yan Shan, Yuhai Wang, Changlei Liang

**Affiliations:** a Department of Cadre’s Ward, The 904th Hospital of Joint Logistic Support Force, Wuxi, China; b Department of Neurosurgery, The 904^th^ Hospital of Joint Logistic Support Force, Wuxi, China.

**Keywords:** outcome, RCT, RIPC, traumatic brain injury

## Abstract

**Background:**

Traumatic brain injury (TBI) is a significant contributor to global mortality and impairment. Experimental data has shown the advantages of remote ischemic preconditioning (RIPC) in treating brain injury, however, there is a lack of evidence-based medicine regarding its clinical effectiveness and safety.

**Materials and methods:**

In this study, we investigated whether RIPC could enhance outcomes in patients with severe TBI. Between January 2019 and December 2022, a comprehensive assessment was conducted on 392 individuals with severe TBI. Out of these, 304 patients were initially included and randomly assigned to receive either RIPC treatment (n = 153) or a control treatment (n = 151). The main measures of results included Glasgow Outcome Scale scores at 6 months, the occurrence of cerebral infarction during hospitalization, mortality rate within 30 days, levels of neuron-specific enolase and S-100β, any adverse effects, expenses incurred during hospitalization, and duration of hospital stay.

**Results:**

The 2 groups did not show any statistically significant differences in baseline clinical data. The Glasgow Outcome Scale scores at 6 months in the RIPC group showed significant improvement when compared with the control group. Additionally, the application of RIPC therapy can reduce the concentrations of neuron-specific enolase and S-100β. There was no notable distinction observed between the 2 groups regarding the adverse reactions of RIPC-induced objective indications of tissue or neurovascular harm. In the RIPC group, there was a significant reduction in both the duration of hospital stays and the expenses associated with hospitalization.

**Conclusion:**

The results of this study suggest that RIPC has the potential to enhance clinical outcomes, mitigate nerve damage, and reduce both hospital expenses and length of stay in patients with severe TBI. The use of RIPC is a reliable and efficient method for managing severe TBI.

## 1. Introduction

It is indisputable that traumatic brain injuries (TBIs) are among the primary factors contributing to fatalities and impairments worldwide.^[1,2]^ According to Maas et al,^[3]^ the number of individuals experiencing TBI annually exceeds 50 million, with an estimated occurrence of one or more TBIs throughout their lifetime for about half of the global population. Similar findings were observed in a recent study that involved 7145 individuals with TBI who received treatment at 47 hospitals in China.^[4]^ Not only does a head injury inflict both physical and mental distress upon the patient, but it also imposes a significant economic burden on both their families and society at large. Despite the significant decrease in the mortality rate of patients with severe TBI^[1,5,6]^ due to enhanced critical management and the utilization of multimodal monitoring technology, there remains a lack of effective treatment for the fundamental pathophysiology of TBI.

The initial discovery of ischemic preconditioning (IPC) was made by Murry et al in 1986,^[7]^ it refers to the repetitive occurrence of temporary myocardial ischemia, which effectively minimizes the subsequent harm caused by long-term myocardial ischemia-reperfusion. Further research has indicated that ischemic preconditioning can decrease the occurrence and intensity of arrhythmia following ischemia, enhance the recovery of cardiac function after complete cardiac ischemia, and lower the likelihood of ischemia-reperfusion injury.^[8–10]^ According to a study by Masada et al,^[11]^ IPC technology has the potential to reduce brain swelling, maintain the integrity of the blood-brain barrier, improve neurological impairments, and minimize the size of the infarcted area following an ischemic stroke. According to Saber and colleagues,^[12]^ it was also found that mice subjected to remote ischemic conditioning experienced reduced acute lung damage following TBI. Numerous fundamental research studies have validated the significant neuroprotective benefits of IPC in diseases of the central nervous system (CNS).^[7–12]^ In clinical practice, it faces a great deal of adjustments, as the heart and brain are very sensitive to ischemic injury. It is difficult to perform ischemic preconditioning directly on the brain or heart. Additionally, the specific efficacy and safety remain undetermined. Hence, the novel idea of remote ischemic preconditioning (RIPC) involves inducing temporary ischemic prestimulation in distant body parts like limbs to enhance the resilience of vital target organs against subsequent severe ischemic damage. This technique serves as an efficient approach to safeguard organs and tissues from the impact of ischemia-reperfusion. In a randomized trial, it was shown that the RIPC method can effectively lower levels of intraoperative troponin I (cTnI) and reduce the occurrence of myocardial injury, myocardial infarction, and renal impairment.^[13]^ In a recent study, it was discovered that preoperative RIPC has the potential to decrease nerve damage and the secretion of Neuron Specific Enolase (NSE) and S-100β following decompression surgery among patients with cervical spondylotic myelopathy.^[14]^ NSE and S-100β were 2 important biomarkers of brain damage.^[15–17]^ TBI causes cerebral hypoxia-ischemia, which is crucial in producing numerous severe secondary brain injuries. RIPC could potentially enhance outcomes in cases of brain injuries to the CNS, as the mechanisms are similar. To date, no high-quality prospective randomized controlled studies examining the clinical significance and safety of RIPC in patients with TBI.

Nevertheless, due to the distinctive characteristics of individuals with TBI and the absence of medical research supported by substantial sample sizes, the effectiveness of RIPC remains uncertain. Therefore, the current investigation aimed to examine the theory that the application of preoperative RIPC could enhance the outcomes in patients with severe TBI.

## 2. Methods

### 2.1. Study design

**Figure 1:**
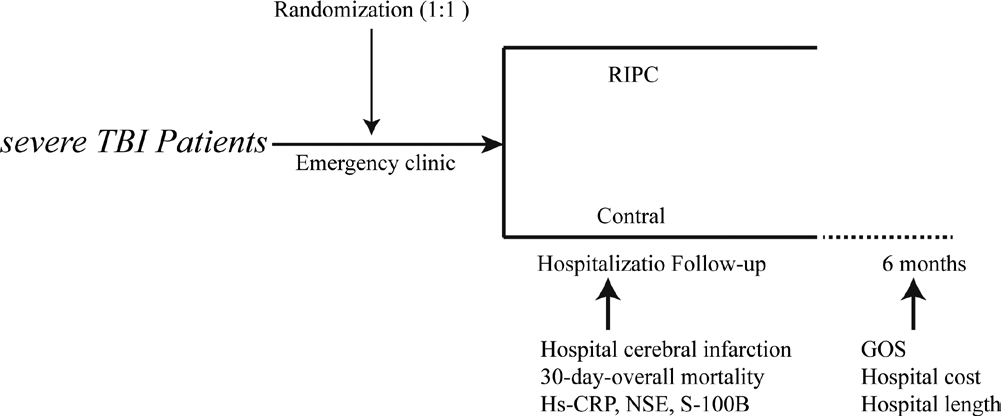
Study design.

From January 2019 to December 2022, a randomized trial with parallel arms was conducted at Wuxi Taihu Hospital in Jiangsu, China. This study adhered to the guidelines provided by the Consolidated Standards of Reporting Trials (CONSORT).^[18]^ During this period, screening was conducted on a total of 392 patients with severe TBI, out of which 304 were initially enrolled in the study. The methodology employed in the current research (2013-YXLL-101) was approved by the Wuxi Taihu Hospital Clinical Research Ethics Committees and adhered to the principles outlined in the Declaration of Helsinki. The registration number was ChiCTR1800015515 (date: April 4, 2019). The study required immediate family members of the patients to acquire written informed consent. Patients were allocated by randomization (1:1) to the intervention or control group. Furthermore, individuals in the RIPC category experienced 4 sets of 5-minute periods of ischemia followed by 5-minute periods of reperfusion on a single upper limb. The nurse anesthetist utilized an electronic tourniquet device (Tourniquet 4500 ECL, manufactured by VBM Medizintechnik, Sulz am Neckar, Germany) to carry out the procedure. Inflating the tourniquet to 200 mmHg to achieve an ischemic effect, and keep 5 minutes, then deflating it and reperfusion it for 5 minutes. Inflating the tourniquet 15 mm Hg above the patient’s systolic blood pressure occurred whenever the patient’s systolic blood pressure surpassed 185 mm Hg. The intervention took place in both the emergency clinic and operating room, concluding with the final round of ischemia and reperfusion. The specific methods of RIPC were performed according to a previous study.^[19]^ The patients in the control groups were provided with regular care before, during, and after the surgery. Figure 1 provides a visual representation of the patient flow details. The final examination took place half 6 months after the operation.

## 3. Patients enrolled in the study and sample selection procedures

The current study included individuals in the emergency intensive care unit. The study staff approached the participants, assessed their eligibility, acquired informed consent, and enrolled them. The inclusion criteria were as follows: (1) individuals between the ages of 18 and 65; (2) confirmed diagnosis of severe TBI through CT or MRI within 8 hours of the trauma; (3) patients requiring immediate craniotomy as evaluated by 2 neurosurgeons; (4) randomly assigned to either receive RIPC or serve as a control group; and (5) preoperative Glasgow Coma Scale (GCS) score ranging from 5 to 8 points. The exclusion criteria included: (1) patients who were not expected to recover upon admission; (2) individuals younger than 18 or older than 65 years; (3) GCS score of 3–4 or >8; (4) pregnant women and patients; (5) those with severe cervical, chest, and abdominal injuries; (6) individuals with multiple organ dysfunction; and (7) any other explanations identified by the researchers.

## 4. Randomization and concealment

Using the SPSS software (version 20.0), we conducted permuted-block randomization. This process involved a computer system that utilized an allotment list to generate random numbers in a 1:1 ratio. The block is set as 2. To preserve the research integrity and blinding, a statistician who was not part of the research team conducted the task. The results of the arbitrary sampling procedure were placed in prenumbered envelopes and stored at the research site until the completion of the study. Neither the research participants nor the patients/family members knew which treatment was being administered during the trial. Every single instance was meticulously documented. Next, we gathered data regarding the patient’s characteristics, past medical records, and relevant examination results.

## 5. Blood samples and plasma testing

A preoperative sample and a postoperative sample were taken from the cubital vein. The preoperative sampling time was upon admission, and the postoperative sampling time was 2 days after the operation. Immediately after the collection of these samples, they were promptly subjected to centrifugation. Fresh plasma samples were used to measure NSE levels, whereas samples stored at − 80°C were used to measure plasma S-100β levels until batch evaluation. An enzyme-linked immunosorbent assay (ELISA) detection kit was utilized to measure the levels of NSE and S-100β. The specific method is based on previous research in our lab.^[20]^

## 6. Outcome assessment

A masked independent diagnostic and assessment committee evaluated all clinical and imaging data as well as the treatment. The committee consisted of 2 researchers who received training before the commencement of the current study and were not involved in the provision of clinical care to patients. The primary endpoint of this research was the 6-month Glasgow Outcome Scale (GOS), which categorized the results into 5 levels: grade 5 indicated a positive recovery, grade 4 indicated a minor disability, grade 3 indicated a severe disability, grade 2 indicated a continuous vegetative state, and grade 1 indicated death.^[1,21]^ Additionally, it was categorized into 2 groups: positive (GOS 4–5) and negative (GOS 1–3). The secondary endpoints consisted of (1) hospital cerebral infarction ascertained by CT and/or MRI,^[2]^ 30-day overall mortality,^[3]^ NSE levels and plasma S-100β levels were measured.

## 7. Assessment of safety and potential issues

We monitored the duration of stay in the intensive care unit, and the primary negative outcomes of RIPC consisted of observable indications of tissue or neurovascular harm. After 2 doctors and nurses who were unaware of the study protocol conducted a physical examination, they confirmed and documented all complications. The examination included checking the distal radial pulses through palpation, visually inspecting for local edema, erythema, and skin lesions, and palpating for tenderness. In the first 14 days, we diligently examined the associated difficulties daily.

## 8. Hospital stays after surgery and the expenses associated with hospitalization

Patients with severe TBI usually stay in the hospital for a long time, and the hospitalization cost is large. If complications and prognosis are improved, postoperative hospital stays and hospitalization costs would decrease significantly. So, postoperative hospital stays and hospitalization costs were important assessment criteria. This study investigated the disparity in the total length of hospitalization, duration of ICU stay, and healthcare expenses between patients from the 2 groups.

## 9. Estimations of the size of the sample

During our initial trial, the main objective indicated that the positive outcome was 58% (18/31) among the RIPC group, whereas it was 43% (15/35) in the control group. The size of the sample was determined based on a significance level of 0.05, a statistical power of 80%, and a 10% attrition rate. Subsequently, a total of 294 individuals were registered, with an equal distribution of 147 participants in both groups.

## 10. Statistical analysis

The means with standard deviations were used to express continuous variables. Statistical analyses were conducted using SPSS software (version 20.0). All data were checked by the data Committee. Quantitative data was assessed using independent-sample t-tests. The chi-square test or Fisher exact t-test was used to compare qualitative data. Statistical significance was determined when the value of P was <0.05.

## 11. Results

### 11.1. Clinical data and baseline assessment

**Figure 2:**
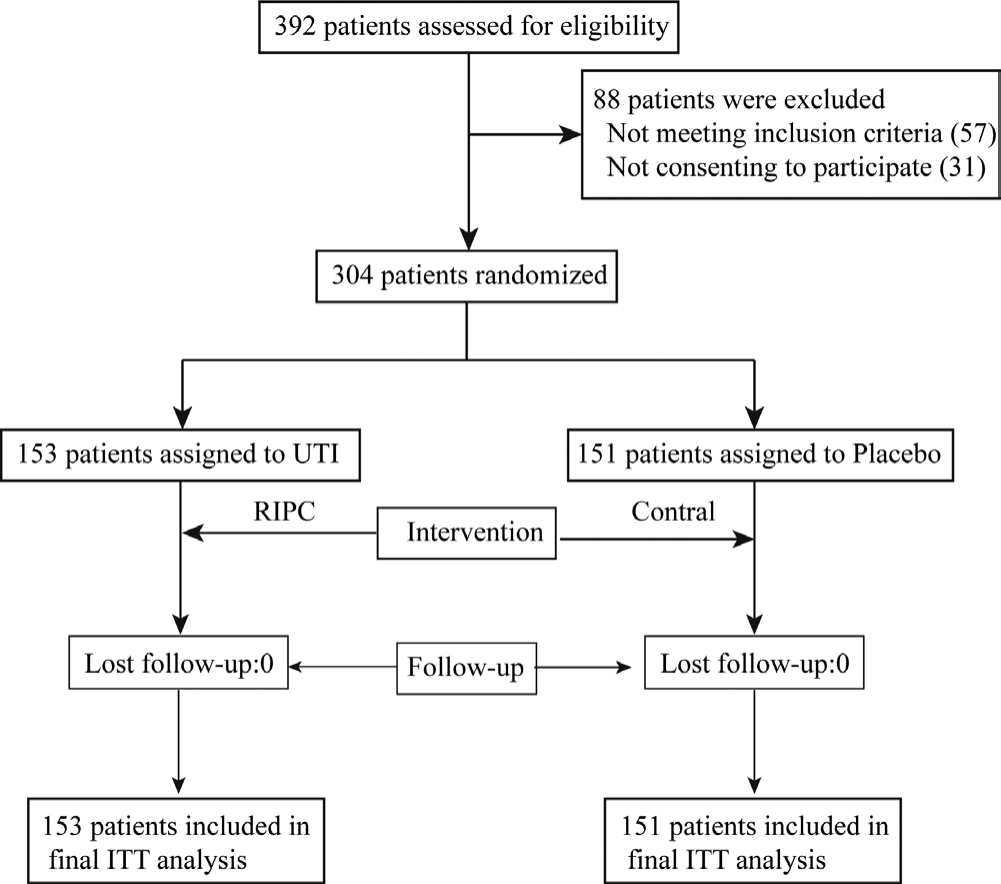
Trial profile.

Between January 2019 and December 2022, a comprehensive assessment was conducted on 392 individuals with severe TBI. Out of these, 304 patients were initially included and randomly assigned to receive either RIPC treatment (n = 153) or a control treatment (n = 151). No instances of visible blindness were observed during the duration of the study. Moreover, there were no statistically significant disparities found in the baseline information between the 2 groups (Table 1). No patients were lost during the entire duration of the current study. All patients were included in the eventual intention-to-treat analysis, as shown in Figure 2. The last patient and final follow-up on May 31, 2023.

**Table 1:** Comparison of baseline data.

	RIPC group (n = 153)	Control group (n = 151)	*P*
Age (Y, mean ± SD)	48.2 ± 11.5	49.1 ± 12.3	0.510
Gender, no. (%)			0.493
Male	102 (66.67%)	95 (62.91%)	
Female	51 (33.33%)	56 (37.09%)	
BMI (KG/cm^2^, mean ± SD)	21.8 ± 1.9	22.1 ± 2.1	0.192
GCS at admission			0.608
3–5	57 (37.25%)	52 (34.44%)	
6–8	96 (62.75%)	99 (65.56%)	
Intubation	41 (26.80%)	37 (24.50%)	0.647
Time (from TBI to surgery)	9.8 ± 4.3	9.2 ± 3.9	0.204
Mydriasis			0.939
No	49 (32.03%)	45 (29.80%)	
Single	58 (37.91%)	63 (41.72%)	
Bilateral	46 (30.06%)	43 (28.48%)	
Rotterdam CT score at admission			0.908
I–II	29 (18.96%)	32 (21.19%)	
III–IV	62 (40.52%)	54 (35.76%)	
V–VI	62 (40.52%)	65 (43.05%)	
Smoking history, no. (%)			0.404
Yes	67 (43.79%)	59 (39.07%)	
No	86 (56.21%)	92 (60.93%)	
Living environment, no. (%)			0.571
Town	68 (44.44%)	72 (47.06%)	
Countryside	85 (55.56%)	79 (52.94%)	
Past medical history, no. (%)			
Hypertension	52 (33.99%)	59 (39.07%)	0.357
Hyperlipidemia	63 (41.18%)	67 (44.37%)	0.574
Diabetes	41 (26.80%)	37 (24.50%)	0.647
Type of hematoma, no. (%)			0.786
Epidural	47 (30.72%)	42 (27.81%)	
Subdural	72 (47.06%)	68 (45.03%)	
Intracerebral	84 (54.90%)	81 (53.64%)	
Surgery time (hours, mean ± SD)	2.1 ± 0.8	2.3 ± 1.1	0.07

BMI = body mass index, CT = computerized tomography, Glasgow Coma Scale, SD = standard deviation, TBI = Traumatic brain injury.

## 12. The primary endpoint

**Figure 3:**
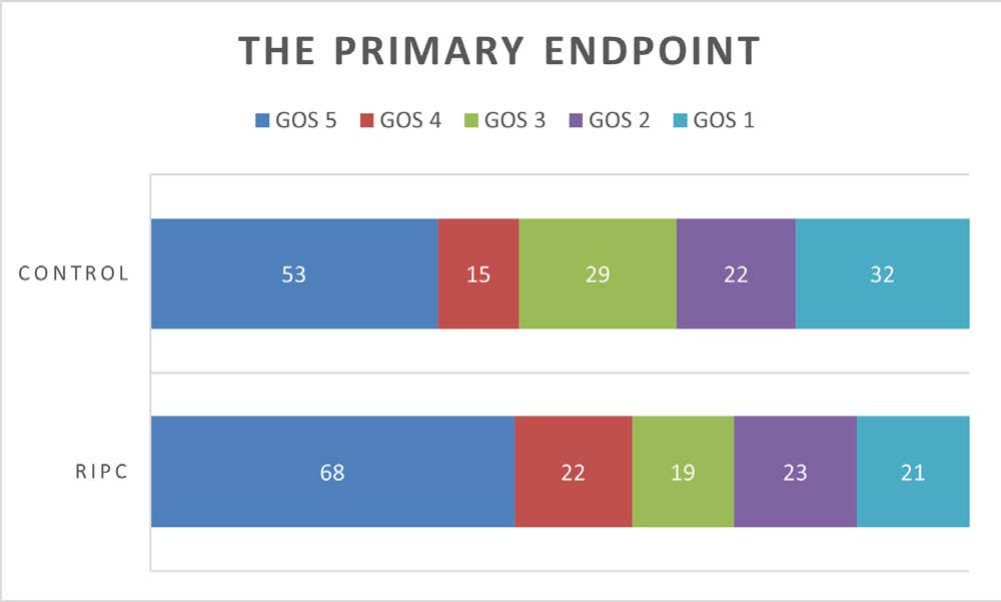
The primary endpoint.

Following 6 months, notable variations in GOS categorization were observed between the 2 groups (*P* = .037, as shown in Table 2 and Figure 3). Patients in the RICP group had a greater proportion of good recovery (58.82%, 90/153) compared with the control group (45.03%, 68/151). During the study, a total of 21 patients (13.73%) from the RICP group and 32 patients (21.19%) died.

**Table 2:** Comparison of the primary endpoint.

	RIPC group (n = 153)	Control group (n = 151)	P
GOS			0.037
Good recovery	68 (44.44%)	53 (35.10%)	
Moderate disability	22 (14.38%)	15 (9.93%)	
Severe disability	19 (12.42%)	29 (19.21%)	
Vegetative state	23 (15.03%)	22 (14.57%)	
Dead	21 (13.73%)	32 (21.19%)	

GOS = Glasgow outcome scale.

## 13. The secondary endpoints

Following RICP therapy, the NSE concentrations (34.18 ± 7.62 vs 37.81 ± 8.19, *P* < .001) and plasma S-100β levels (1529.14 ± 338.08 vs 1726.14 ± 342.15, *P* < .001) exhibited notable enhancements in the RICP group when compared with the control group (Table 3). There was no statistically significant difference (*P* > .05, Table 3) in the occurrence of cerebral infarction and 30-day overall mortality between the 2 groups.

**Table 3:** Comparison of the secondary endpoints.

	RIPC group (n = 153)	Control group (n = 151)	*P*
NSE levels(ng/mL)	34.18 ± 7.62	37.81 ± 8.19	<0.001
S-100β(ng/L)	1529.14 ± 338.08	1726.14 ± 342.15	<0.001
Cerebral infarction	8 (5.23%)	15 (9.93%)	0.121
30-day-overall mortality	18 (11.76%)	26 (17.22%)	0.177

NSE = neuron-specific enolase.

## 14. Safety evaluation

Objective indications of tissue or neurovascular harm were the most common negative consequences of RIPC. At the location where the cuff was positioned, 3 individuals encountered a hematoma in their lower extremities, with no observed reduction in hemoglobin levels. After undergoing RIPC, 9 patients experienced temporary cyanosis, while 5 patients experienced temporary erythema; there were no cases of acute limb ischemia observed. Table 4 shows that the RIPC group had 9 patients with deep vein thrombosis, while the control group had 6 patients. There was no notable distinction found between the 2 groups regarding the potential adverse reactions.

**Table 4:** Comparison of the safety evaluation.

	RIPC group (n = 153)	Control group (n = 151)	*P*
Hematoma in lower limb	3 (1.96%)	NA	NA
Cyanosis in lower limb	9 (5.88%)	NA	NA
Erythema in lower limb	5 (3.27%)	NA	NA
Deep vein thrombosis	9 (5.88%)	6 (17.22%)	

## 15. Postoperative hospital stays and hospitalization costs

The RIPC group had an average stay of 22.16 days, while the control group had a duration of 23.78 days, and this disparity was found to be statistically significant (*P* = .018). The average hospitalization cost for the RIPC group was 84,900 RMB, significantly lower than the control group’s expenditure of 91,600 RMB (*P* = .017, Table 5).

**Table 5:** Comparison of Postoperative hospital stays and costs.

	RIPC (n = 153)	Control (n = 151)	*P*
Hospitalization stays, day, mean ± SD	22.16 ± 5.74	23.78 ± 6.12	0.018
Hospitalization costs, CNY*10^4^, mean ± SD	8.49 ± 2.28	9.16 ± 2.57	0.017

CNY = Chinese Yuan, SD = standard deviation.

## 16. Discussion

Based on the results of the present study, remote ischemic preconditioning (RIPC) has the potential to significantly enhance the 6-month prognosis for individuals with severe TBI. Furthermore, we discovered that RIPC can reduce NSE levels and plasma S-100β levels, which serve as indicators of nerve injury. Moreover, it resulted in a decrease in both the duration of hospitalization and the associated costs. Multiple studies have indicated that in patients with TBI, the levels of serum NSE were found to have a significant inverse correlation with GCS scores, this suggests that it can serve as a crucial indicator for assessing brain damage.^[15,16]^ Furthermore, there was no substantial rise in the frequency of severe adverse reactions.

RIPC involves intermittent ischemia and reperfusion of the distal organ to protect the target vital organ from subsequent lethal ischemia-reperfusion injury, and several organs and illnesses have been tested for RIPC.^[22,23]^ Several preclinical and clinical investigations have shown that RIPC protects the organ.^[22–24]^ The exact process through which RIPC provides organ protection is not well comprehended; nevertheless, it has been demonstrated to play a role in neural, humoral, and immunological pathways. After undergoing the RIPC procedure, the group of rats that received treatment showed better performance in the water maze test in comparison to the group that did not receive any treatment. Furthermore, the immunostaining analysis demonstrated a decrease in the amount of hippocampal area nerve cell fatalities.^[25]^ A recent study has shown that the TNF-related apoptosis-inducing ligand T receptor is vital in causing the death of nerve cells. The expression of this receptor can be suppressed by RIPC, resulting in a reduction in both brain infarct volume and neuronal mortality.^[26]^ Prior research has indicated that the neuroprotective mechanisms of ischemic preconditioning might encompass the subsequent. First, RIPC locally produces substances that have a protective effect by activating neural pathways that reach brain tissue and then activate receptors within the brain tissue.^[27,28]^ Second, local chemicals or enzyme-linked reactions are generated during ischemia and reperfusion of distal organs, which subsequently traverse the circulatory pathway to activate receptors and facilitate signal transduction, ultimately mediating neuroprotective effects.^[29]^ Animal studies conducted previously have also suggested that RIPC can control inflammation,^[30]^ reduce oxidative stress,^[31]^ induce apoptosis,^[32]^ and provide neuroprotection.^[32–34]^ Organ protection is believed to depend on the intricate interaction among these pathways.

TBI, one of the most crucial issues in public health, has a profound impact on the lives of those who are injured and their families, and it is linked to a considerable rate of death.^[1,21]^ Neurologic deficits are linked to TBIs due to the death of neurons, making neurons a viable target for therapy.^[35]^ Multiple studies have indicated that the use of drugs or RIPC to suppress apoptosis, ferroptosis, oxidative stress, neuroinflammation, necrosis, necroptosis, and autophagy can mitigate initial brain damage following TBI or other acute CNS disorders.^[20,32,33,35–37]^ According to Saber et al,^[12]^ RIPC may be advantageous for managing TBI-induced acute lung injury by preserving lung function and aiding in clinical management. According to Wu, et al,^[38]^ it was also found that the impact of hypoxia preconditioning on hypoxia tolerance and neuronal damage in the cerebral cortex of rats after TBI can be reduced. This reduction is primarily associated with the enhancement of glucose transportation activity by inducing the expression of GLUT1 and GLUT3. Furthermore, Benitez et al^[39]^ discovered that hypoxic-ischemic insult affects glutamatergic and GABAergic neurons in the cerebellum of neonatal rats, leading to their impairment. However, preconditioning hypoxia offers protection specifically to the former. Therefore, sporadic ischemic therapy of the peripheral limb or other body regions may safeguard the brain against ischemia-reperfusion damage. The mechanism of action for RIPC remains uncertain, despite the involvement of multiple pathways, factors, and molecules in its formation. Animal experiments have shown the notable clinical impacts of RIPC, while human trials, particularly in patients with TBI, are yet to commence. Crucially, this study marks the initial validation of a randomized controlled trial conducted at a single center with a large sample size to verify the clinical significance of RIPC in patients with severe TBI. The findings demonstrate that RIPC can enhance the 6-month outcomes following craniotomy in severe TBI patients, mitigate nerve damage, and reduce both hospital costs and length of stay.

Here are a few of the constraints that were identified in the study. The findings may not be extrapolated as this research was a trial that was randomized and controlled, conducted at a single center. Another constraint is that we solely conducted a follow-up for 6 months, potentially necessitating an evaluation of long-term results. The study is limited by the small number of participants. To further investigate the clinical outcome of RIPC treatment in severe TBI patients, additional multicenter randomized controlled trials will be required in the future.

## 17. Conclusion

Our research found that implementing RIPC as a treatment method, specifically during craniotomy therapy for patients with severe TBI, resulted in enhanced outcomes at the 6-month mark, reduced nerve damage, and lowered both hospital expenses and length of stay. This finding provides evidence to support the execution of a substantial randomized clinical trial, incorporating a practical primary measure, for the implementation of RIPC therapy upon hospital admission for individuals in the early stage of TBI.

## Author contributions

**Conceptualization:** Yuhai Wang, Changlei Liang.

**Data curation:** Juan Shen, Yan Shan, Yuhai Wang.

**Formal analysis:** Lin Zhu, Yan Shan.

**Investigation:** Juan Shen, Lin Zhu, Changlei Liang.

**Methodology:** Juan Shen, Lin Zhu, Yan Shan, Changlei Liang.

**Project administration:** Changlei Liang.

**Resources:** Yan Shan.

**Software:** Changlei Liang.

**Supervision:** Juan Shen, Yan Shan, Changlei Liang.

**Validation:** Yan Shan, Changlei Liang.

**Visualization:** Juan Shen, Lin Zhu, Yan Shan, Changlei Liang.

**Writing—original draft:** Juan Shen, Lin Zhu, Yan Shan, Yuhai Wang, Changlei Liang.

**Writing—review and editing:** Juan Shen, Changlei Liang.
